# Spironolactone Inhibits NADPH Oxidase-Induced Oxidative Stress and Enhances eNOS in Human Endothelial Cells

**Published:** 2011

**Authors:** Ashraf Taye, Henning Morawietz

**Affiliations:** a *Department of Pharmacology and Toxicology, Faculty of Pharmacy, Minia University, Egypt.*; b *Department of Vascular Endothelium and Microcirculation, Medical Faculty Carl Gustav Carus, University of Technology Dresden, Germany*.

**Keywords:** Aldosterone, Spironolactone, Oxidative stress, NADPH oxidase, Endothelium

## Abstract

Accumulating evidence indicates that aldosterone plays a critical role in the mediation of oxidative stress and vascular damage. NADPH oxidase has been recognized as a major source of oxidative stress in vasculature. However, the relation between NADPH oxidase in aldosterone-mediated oxidative stress in endothelial cells remains to be ascertained. The present study aimed to investigate the relevant role of NADPH oxidase in aldosterone induced oxidative stress and the functional consequence of this effect on endothelial function. Additionally, we attempted to examine the potential role of the mineralocorticoid receptor (MR) antagonist; spironolactone (spiro) in this scenario. Human umbilical artery endothelial cells (HUAECs) were incubated with aldosterone (100 nmol/L, 24 h) in the absence and presence of Spiro (1 µmol/L). The results showed that aldosterone significantly increased the protein expression of NADPH oxidase subunits (Nox2, p47^phox^ and p22^phox^) and that spiro markedly inhibited these changes. Functionally, this was associated with an elevation in NADPH oxidase activity and 3-nitrotyrosine (3-NT) as a biochemical marker of oxidative stress. However, pre-incubation with spiro inhibited these consequences. Moreover, MR protein expression was upregulated by aldosterone whereas this effect was suppressed by Spiro. While aldosterone effectively inhibited endothelial nitric oxide (eNOS) protein expression, pretreatment with spiro markedly restored it to its normal level. In conclusion, the results achieved suggest that aldosterone may play a critical role in NADPH oxidase-mediated oxidative stress resulting in reduced eNOS expression in human endothelial cells. Spiro effectively reversed these consequences, suggesting its potential vasculoprotective effect in endothelial dysfunction.

## Introduction

Oxidative stress contributes to increased cardiovascular morbidity and mortality associated with the activation of the renin-angiotensin-aldosterone system. Studies in cultured cells *in-vitro *(1) and in rodent models *in-vivo* (2) demonstrated that aldosteroneand/or mineralocorticoid receptor (MR) activation causes oxidativestress in addition to vascular inflammation. Similar to angiotensinII, aldosterone can also cause endothelial dysfunction (3, 4).

Several mechanisms account for the deleterious effects of aldosterone on endothelium with one important effect being a decrease in NO availability. This decrease may not only involve a reduction in NO production but also an increase in NO inactivation by reactive oxygen species (5, 6). Aldosterone can increase oxidative stress by both increasing reactive oxygen species (ROS) production and reducing the ROS scavenging capacity of the cells. Aldosterone administration in uninephrectomized rats treated for 4 weeks with dietary 1% NaCl increased H_2_O_2_ production by monocytes and lymphocytes (7). Similarly, aldosterone administration increased vascular superoxide production in normal rats (8). Aldosterone-induced ROS has been observed in different pathological situations in hypertensive animals (9) as well as in two different models of atherosclerosis (10).

On the other hand, several molecular sources of endothelial ROS formation have been suggested (11, 12). NADPH oxidase complexes have been recognized as a major source of superoxide anions in endothelial cells (13-15). Thees NADPH oxidase complexes contain different catalytic NADPH oxidase subunits (16). Up to seven NADPH oxidase isoforms have been described in different cell types (17). The classical NADPH oxidase complex is composed of a membrane bound flavocytochrome b_558_ consisting of gp91^phox ^(Nox2) and p22^phox^ as well as cytosolic subunits (13, 14). Besides the gp91^phox^/Nox2-containing complex, Nox4 is the prominent isoform in endothelial cells (14, 18). Nox2 is principally located in the endothelium and adventitia and it is essential for the activation of this enzyme in response to angiotensin II (14). 

Although aldosterone plays a crucial role in vascular oxidative stress, its potential mechanism with respect to NADPH oxidase activation in endothelial cells still remains to be addressed. The present study was designed to identify the relevant role of NADPH oxidase in aldosterone-induced oxidative stress and the functional consequence of this effect regarding endothelial function. Additionally, we attempted to examine the potential role of the MR antagonist; spironolactone (spiro) in this setting.

## Experimental

***Cell Culture***

All cell culture reagents and chemicals were purchased from Sigma Chemical Co., unless indicated otherwise. Primary cultures of HUAECs were isolated with collagenase IV and cultured in M199 medium (Life Technologies) supplemented with 20% (v/v) calf serum, as previously described (19) Confluent cell cultures were incubated with endothelium medium with a low calf serum 0.5% (v/v) for 24 h.These were subsequently treated with aldosterone (100 nmol/L) in the absence or presence of the MR antagonist, spiro (1 µmol/L). 

**Estimation of NADPH oxidase activity**

NADPH oxidase activity was measured in cells by using lucigenin-derived chemiluminescence as described previously (20). The formation of a reactive oxygen species in response to aldosterone (100 nmol/L) was analyzed in HUAEC. The endothelial cells were detached, adjusted and incubated in white 96-well using trypsin (21). Subsequently, cells were transferred to a Krebs–Henseleit solution (10 mmol/L glucose, 0.02 mmol/L Ca-Tritriplex, 25  mmol/L NaHCO_3_, 1.2  mmol/L KH_2_PO_4_, 120  mmol/L NaCl, 1.6  mmol/L CaCl_2_·2H_2_O, 1.2  mmol/L MgSO_4_·7H_2_O, and 5 mmol/L KCl, pH 7.4). Oxygenradical production was measured in the absence and presence of lucigenin (5 µmol/L), NADPH (100 µmol/L) , apocynin (NADPH oxidase inhibitor; 10 µmol/L), uncoupled eNOS inhibitor, **N**^ω^-nitro-L-arginine methyl ester (L-NAME; 10 µmol/L) and allopurinol (xanthine oxidase inhibitor; 10 µmol/L), and Spiro (1 µmol/L) for 20 min using a FLUOstar OPTIMA multi-well reader (BMG, Offenburg, Germany). Results are expressed as relative light units per minute per 10^5^ cells. In another set of experiments, the antioxidant properties of spiro were exa,ined using a xanthine/ xanthine oxidase assay. 500 µmol/L of xanthine was added to 10 mU/mL of xanthine oxidase in the absence and presence of a western lightening reagent. Then resulting chemiluminescence was measured in the absence and presence of a concentration-dependent (0.1, 1, 10 µmol/L) spiro. The validity of the assay was performed using a membrane-permeable superoxide dismutase (SOD) mimetic.

*Western blot analysis*

Cells were lysed in ice-cold lysis buffer containing the following: 20 mmol/L Tris· HCl, 140 mmol/L NaCl, 1 mmol/L EDTA, complete miniprotease inhibitor cocktail, 1% Triton X-100, 0.1% SDS (Sodium Dodecyl Sulfate),1% sodium deoxycholate, 1 mmol/L NaF, and 1 mmol/L orthovanadate, pH7.8. The protein concentration was determined using the BCA protein assay reagent (Perbio Science, Bonn, Germany). Equal amounts of membrane protein (20 µg/lane)were separated by sodium dodecyl sulfate–polyacrylamidegel electrophoresis (SDS-PAGE) and transferred to polyvinylidene fluoride membranes (Roth, Karlsruhe, Germany). After incubation in blocking solution (4% nonfat milk, Sigma), membranes were incubated with 1: 1000 Nox4, p22^phox^, p47^phox^ and rabbit polyclonal antibody *against* MR (1:300) (Santa Cruz), Nox2 (upstate Lab), eNOS and 3- nitrotyrosine (3-NT) antibodies for 2 hours at room temperature. Membranes were washed and then incubated with a 1:3000 dilution of the second antibody (AmershamLife Science) for 1 h. The membranes were then detected withthe enhanced chemilumine scence system (amersham life science).To correct for differences in protein loading, the membranes were washed and reprobed with a 1:1000 dilution of monoclonal antibody to human *ß*-actin (abcam). The relative intensities of the protein bands were analyzed by a scanner (model Scanmaker 8700, Microtek Laboratory) (22).

*Statistical analysis*

Data are expressed as means ± SEM. A one-way analysis of variance (ANOVA) followed by Tukey’s post test was used to assess the presence of significant differences (p < 0.05). All the statistical analyses were accomplished, using the computer software GraphPad Prism 4 for Windows (GraphPad Software, USA).

## Results

*Effect of aldosterone on NADPH Oxidase, 3-NT and eNOS expressions*

**Figure 1:**
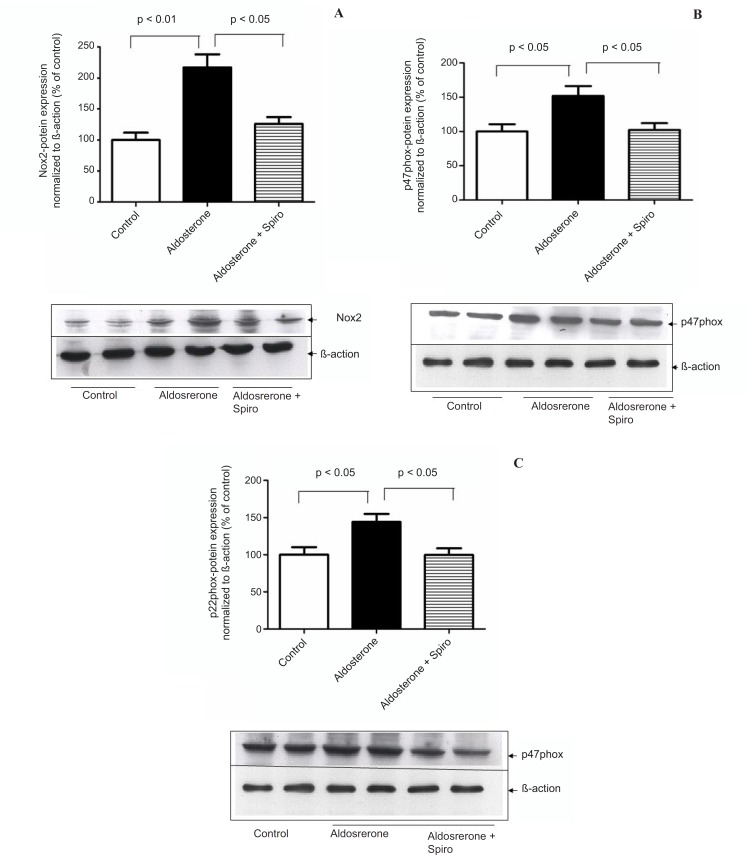
Effects of aldosterone (100 nmol/L) on Nox2 , p47 phox and p22 phox protein levels in HUAEC_S_ incubated for 24 h in the absence or presence of spiro (1µmol/L). (A) Aldosterone significantly increased Nox2 protein expression and spiro markedly inhibited upregulation of the Nox2 protein level. Quantitative analysis of Nox2 protein expression, normalized with *B*-actin, by scanning densitometry. (B) Aldosterone induced the p47phox protein level, this was inhibited on pre-treatment with spiro. Quantitative analysis of the p47^phox^ protein, normalized with *B*-actin, respectively, by scanning densitometry. (C) Preincubation of HUACEs with aldosterone resulted in the upregulation of inp22^phox^ protein expression and this was inhibited on pre-treatment with spiro. Quantitative analysis of the p22^phox ^protein, normalized with *B*-actin, respectively, by scanning densitometry. Values for each bar are mean ± SEM from 4 separate experiments and expressed as % of control, p < 0.05, p < 0.01 *versus *control or indicated bar. Spiro: Spironolactone.

HUAECs stimulated by aldosterone (100 nmol/L) for 24 h showed a significant upregulation in NADPH oxidase, Nox2 (p < 0.01, Figure 1A) as well as the cytosolic p47^phox^ (p < 0.05, Figure 1B) and membrane subunits p22^phox ^(p < 0.05, Figure 1C) protein levels in comparison with the control. Pretreatment with spiro (1 µmol/L) significantly (p < 0.05) inhibited these changes. 

**Figure 2:**
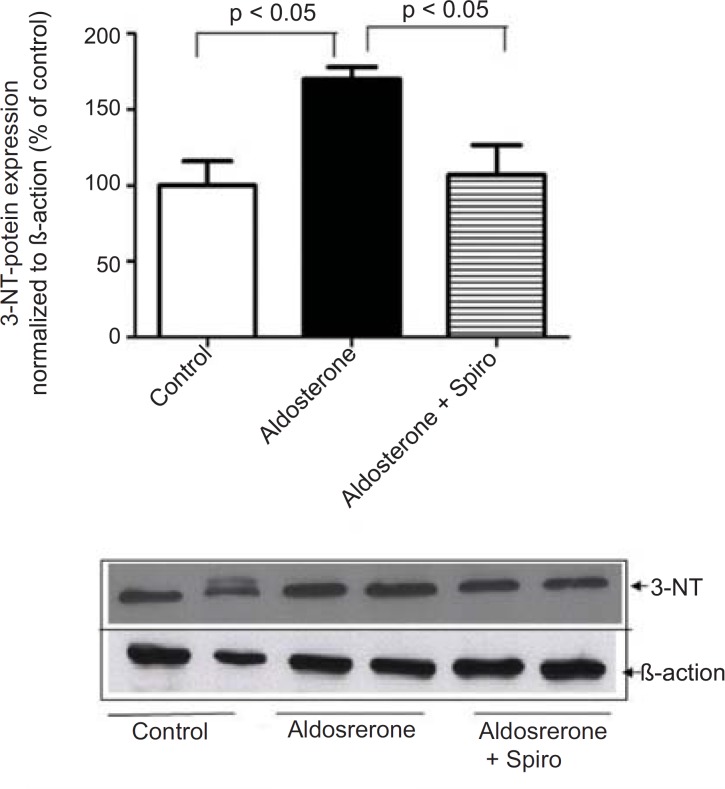
Effects of aldosterone (100 nmol/L) on 3-NT protein levels in HUAECs incubated for 24 h in the absence or presence of spiro (1µmol/L). Aldosterone produced an elevation in 3-NT protein level and spiro (1µmol/L) inhibited. Quantitative analysis of 3-NT protein expression, normalized with *B*-actin, by scanning densitometry. Values for each bar are mean ± SEM from 4 separate experiments and expressed as % of control, p < 0.05 of aldosterones *vs *control and spiro *versus *aldosterone. Spiro: Spironolactone; 3-NT: 3-nitrotyrosine

 Aldosterone did not exhibit any significant changes in the Nox4 protein level. Moreover, stimulation of HUAECs with aldosterone significantly increased the 3-NT protein level as a marker of oxidative stress and this was significantly suppressed by preincubation with spiro (1 µmol/L) (Figure 2). 

**Figure 3:**
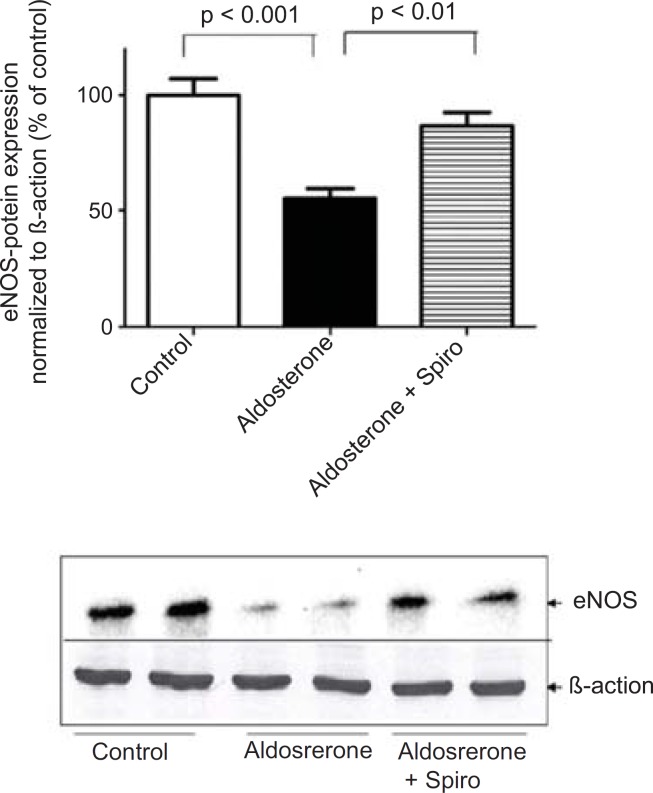
Effects of aldosterone (100 nmol/L) on eNOS protein levels in HUAECs incubated for 24 h in the absence or presence of spiro (1µmol/L). Aldosterone attenuated eNOS protein expression and spiro (1µmol/L) restored in to the normal level was normalized with *B*-actin, by scanning densitometry. Values for each bar are mean ± SEM from 4 separate experiments and expressed as % of control, p < 0.001 of aldosterone *versus *control and p < 0.01 spiro *versus *aldosterone. Spiro: Spironolactone; eNOS: Endothelial nitric oxide synthase.

On the other hand, aldosterone was found to exhibit a marked downregulation in eNOS protein expression and Spiro significantly (p < 0.01) restored it to its normal level (Figure 3).

**Figure 4:**
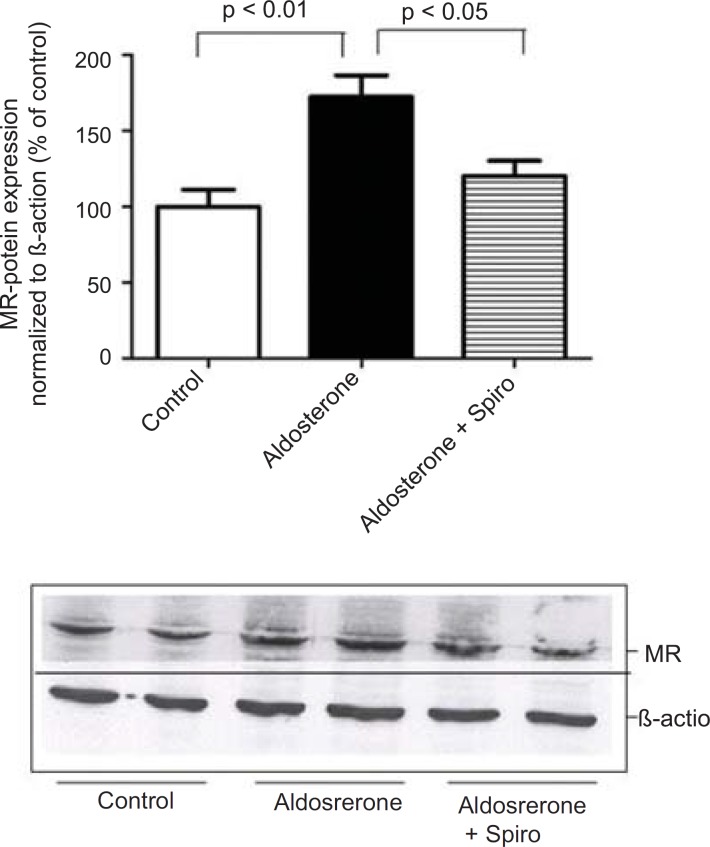
Effects of aldosterone (100 nmol/L) on MR protein levels in HUAECs incubated for 24 h in the absence or presence of spiro (1µmol/L). Preincubation of the HUACEs with aldosterone resulted in an increase MR protein level and spiro (1µmol/L)was able to inhibit the aldosterone-induced MR upregulation. Values for each bar are mean ± SEM from 4 separate experiments and expressed as % of control, p < 0.01 of aldosterone *versus *control and p < 0.05 spiro *versus *aldosterone. Spiro: Spironolactone; MR: Mineralocorticoid receptor.

 Furthermore, preincubation of the HUAECs with aldosterone resulted in an upregulation of MR expression with Spiro significantly inhibiting this response (Figure 4). 

*Effect of aldosterone on the NADPH oxidase activity*

**Figure 5:**
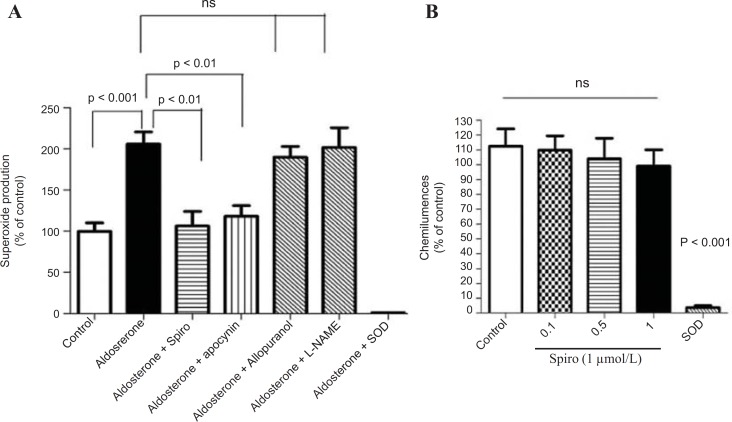
Effect of aldosterone on the NADPH oxidase activity measured by lucigenin-enhanced chemiluminescence. (A) The HUAEC_S_ were prein cubated for 24 h with a vehicle or aldosterone (100nmol/L) in the presence and absence and absence of the MR antagonist , spiro (1µmol/L), apocynin (10µmol/L) , L-NAME (10 µmol/L) and allopurinol (10 µmol/L). Aldosterone (100nmool/L) induces an increase in NADPH oxidase activity measured by lucigenin-enhanced chemiluminescence and this was markedly inhibited by spiro apocynin but not by either L-NAME or allopurinol . (B) xanthine/ xanthine oxidase assay to examine the antioxidant properties of spiro. Spiro could not inhibit the suproxide production generated by the xanthine and xanthine oxidase system. Values for each bar are mean ± SEM from 8 separate experiments and expressed as % of control p < 0.01 and p < 0.001 *versus *control or as indicated bar. Spiro: Spironolactone

The stimulation of the HUAECs with aldosterone (100 nmol/L) for 24 h caused a 2-fold increase in NADPH oxidase activity as measured by lucigenin-derived chemiluminescence (p < 0.001). However, pretreatment with Spiro (1 µmol/L) significantly (p < 0.01) inhibited this effect (Figure 5A). It is noteworthy that the increase in superoxide anion production measured by lucigenin-mediated chemiluminescence was significantly inhibited by apocynin and neither the xanthine oxidase inhibitor nor the uncoupled eNOS inhibitor was able to inhibit the lucigenin enhanced chemiluminescence. SOD was able to completely inhibit the lucigenin-enhanced chemiluminescence, indicating that these chemiluminescences are superoxide anion-dependent. Regarding testing the antioxidant properties of Spiro using xanthine and the xanthine oxidase assay, spiro could not inhibit the suproxide anions generated by xanthine and the xanthine oxidase system. (Figure 5B).

## Discussion

The present study showed that stimulation of the HUAECs with aldosterone resulted in a significant elevation of NADPH oxidase expression and activity. These changes were inhibited by the MR antagonist, spiro. We used a 100 nmol/L dose of aldosterone which is a close approximation to the* in vivo* situation, particularly under hyperaldosteronism conditions. A growing body of evidence indicates that ROS are implicated in many pathophysiological processes including scavenging of endothelium-derived nitric oxide (NO) (23), and prevention of its protective signaling functions (24, 25). Although ROS may derive from mitochondria, xanthine oxidase, cyclooxygenase, uncoupled NO synthase, heme oxygenases, or peroxidases, it has been frequently shown that NADPH oxidasesare the primary producers of ROS in vascular tissues (26, 27).Recent evidence suggests that NADPH oxidases, the only known enzyme family solely dedicated to ROS production, may be a key player. In harmony with this concept, we could not detect any significant role of xanthine oxidase and uncoupled eNOS in total ROS production. As the respective inhibitors, allopurinol and L-NAME, did not affect ROS signals in HUAECs stimulated by aldosterone. In contrast, the NADPH oxidase inhibitor, apocynin reduced ROS formation supporting the hypothesis that NADPH oxidases are indeed a major source of vascular oxidative stress in our system model.

Previous studies have suggested that ROS produced by NADPH oxidase mediate many angiotensin II effects in the cardiovascular system (14, 26). Several reports suggest an important role for aldosterone in the regulation of NADPH oxidase. Recently, it has been reported that aldosterone increases NADPH oxidase expression in the vasculature (28). Systemic administration of aldosterone increases oxidative stress in the heart, vasculature, kidney and increases macrophage NADPH oxidase (29). In addition, MR activation contributes to angiotensin II-mediated activation of NADPH oxidase in the heart and aorta (30). Furthermore, exogenous aldosterone stimulates aortic expression of p22^phox ^and Nox2 through an MR-dependent mechanism and of p47^phox^ mRNA through both an angiotensin type 1 receptor and MR-dependent mechanisms (31). In this regard, the present study demonstrates a marked upregulation of Nox2, in contrast to Nox4 that did not exhibit any change. Accordingly, Nox2 might be considered as the relevant isoform involved in aldosterone-mediated NADPH oxidase activation. On the other hand, the cytosolic component of the p47^phox^ component was shown to havea pivotal role in the regulation of enzymatic activity. It has been reported that the hypertensive response and productionof vascular superoxide was markedly blunted in p47^phox^ knockoutmice (32). It has been also shown that aldosterone induced NADPH oxidase activation and membranous translocation of p47^phox^ in HUAECs (33). In this context, the current study shows that aldosterone increased thep47^phox^ protein level. Thus, aldosterone can increase ROS production in HUAECs by activating NADPH oxidase viap47^phox^ translocational regulation. 

NADPH oxidase catalyzes the one-electron reduction of molecular oxygen to a superoxide anion which can react with nitric oxide to form short-lived peroxynitrite. Peroxynitrite forms stable 3-NT-conjugated protein moieties (24). In this context, aldosterone was found to increase in the 3NT content as a biochemical marker of oxidative stress and this was significantly inhibited with pretreatment using spiro, supporting a potential role of MR in oxidative stress. Spiro was able to inhibit aldosterone-mediated NADPH oxidase activation, suggesting MR might be considered as upstream of NADPH oxidase. Specificity was demonstrated by means of the xanthine/xanthine oxidase assay, where antioxidative effects and flavoenzyme inhibition were excluded. Based on these finding, the present study demonstrates that spiro can antagonize aldosterone-mediated superoxide production and this is attributable to its inhibition of the NADPH oxidase enzyme. It has been reported that superoxide production reduces nitric oxide bioactivity while reducing the expression of nitric oxide synthase (34). Evidence of endothelial dysfunction was seen in isolated renal artery segments and aortic rings from rats exposed to a model of excessive MR stimulation. In animals treated with spiro, normal endothelial function was restored (8, 35). Similarly, in rabbits fed a proatherosclerotic diet, treatment with spiro normalized superoxide formation and improved endothelial function (36). In healthy male volunteers, aldosterone has been shown to cause acute endothelial dysfunction (37). 

Notably, spiro significantly antagonized the inhibitory effect of aldosterone on eNOS expression. Importantly, using MR-reactive antibodies and western blotting we investigated MR protein expression in the HUAECs in response to aldosterone. This suggests that MR was the main receptor mediating the pro-oxidative effect of aldosterone in the current investigations. The present findings are in agreement with the previous *in-vivo *study demonstratingthat eplerenone (a specific MR antagonist) administration to hypercholesterolemic rabbits normalized superoxide generation, decreased NADPH oxidase activity to basal levels and nearly normalized endothelium-dependent vasorelaxation.(10). A recent study showed similar inhibition of atherosclerosis when eplerenone reduced markers of oxidative stress including the ability of macrophages to oxidize low density lipoprotein, macrophage superoxide anion release and the susceptibility of low density lipoprotein to oxidation (38).

Regarding the mechanism of action aldosterone, it has been reported that aldosterone binds intracellular MR and translocates it to the nucleus, where it binds to its ligand and interacts with the regulatory region of target gene promoters (39). In contrast, aldosterone might have a non-genomic effect within minutes (40). Importantly, we could not detect ROS production within 2 h of stimulation by aldosterone, it took much longer as was previously reported (28, 35). However, some reports showed that aldosterone induces ROS production through the activation of NADPH oxidase within 30 min (41, 42). Such a discrepancy might be attributed to using different cell types or different experimental conditions.

In conclusion, the present results demonstrate that aldosterone stimulates NADPH oxidase-mediated oxidative stress thereby reducing eNOS expression. In addition, the MR antagonist, spiro appears to play a potential role in inhibiting these consequences. Thus, the current study suggests that NADPH oxidase might act as key regulator in aldosterone--mediated oxidative stress, thereby reducing eNOS expression. This study adds a new dimension to the understanding of the role of aldosterone in the activation of NADPH oxidase in human endothelial cells and supports the notion that aldosterone can induce the dysregulation of endothelial cells. This knowledge may lead to novel strategies for the prevention of oxidative stress and endothelial dysfunction via the blocking of MR.

## Source of Funding

This study was funded by DAAD (German Academic for Scientific Exchange) and the Ministry of higher education of Egypt.
